# Nomograms for predicting risk of locoregional recurrence and distant metastases for esophageal cancer patients after radical esophagectomy

**DOI:** 10.1186/s12885-018-4796-5

**Published:** 2018-09-10

**Authors:** Wen-Yi Zhang, Xing-Xing Chen, Wen-Hao Chen, Hui Zhang, Chang-Lin Zou

**Affiliations:** Department of Radiotherapy and Chemotherapy, The First Affiliated Hospital of Wenzhou Medical University, WenZhou, China

**Keywords:** Nomogram, Esophageal cancer, Risk prediction, Locoregional recurrence, Distant metastases

## Abstract

**Background:**

The aim of this study was to develop nomograms for predicting the risk of locoregional recurrence or distant metastasis in esophageal cancer patients who were treated with esophagectomy and regional lymphadenectomy.

**Methods:**

The clinicopathologic data of 408 esophageal cancer patients after esophagectomy and regional lymphadenectomy were analyzed in this study. Univariate and multivariate COX regression analyses were used to test the association between the clinicopathologic data and the risk of *locoregional* recurrence or distant metastasis. The nomograms were built from the COX regression model.

**Results:**

Univariate analyses revealed that tumor length, tumor width, T-staging and perineural invasion(PNI) were significantly associated with locoregional recurrence, and that tumor length, tumor width, differentiation, T-staging, N-staging, lymph vascular space invasion(LVSI), PNI and adjuvant chemotherapy were significantly associated with distant metastasis. Multivariate analyses revealed that tumor length, tumor width and T-staging were predictors of risk of locoregional recurrence, and that differentiation, N-staging, LVSI and PNI were predictors of risk of distant metastasis. Two nomograms were constructed for a visual explanation of these two COX regression models. The bias-corrected curve showed no significant departure from the ideal curve in these two nomograms.

**Conclusions:**

Two nomograms were developed and validated to predict the risk of locoregional recurrence and distant metastasis in esophageal cancer patients after radical esophagectomy. The calculation outcome will help oncologists to choose adjuvant treatment regimens.

## Background

Esophagectomy is traditionally considered the standard treatment for esophageal cancer. The median survival time for radical surgery alone rarely exceeds 18 months in most modern series [1].

The role of adjuvant treatments, such as chemotherapy and radiotherapy, for esophageal cancer patients after radical esophagectomy is still controversial. Pasquer A et al. [2] analyzed data from 2944 patients and found that adjuvant chemotherapy or radiotherapy did not offer a survival benefit in esophageal cancer patients with lymph node-positive after surgery. The study by Brescia AA et al. [3] suggested that there was an apparent survival benefit in esophageal cancer patients receiving adjuvant therapy, including chemotherapy, radiotherapy or both, after induction therapy and surgery, especially in patients with 4 or more lymph node positive. Bédard EL and colleagues [4] reviewed patients with resected esophageal carcinoma and found that concurrent or sequential postoperative radiotherapy and chemotherapy appeared to prolong survival in patients with positive lymph nodes. Rice TW et al. [5] concluded that locoregionally advanced esophageal carcinoma patients receiving postoperative adjuvant chemoradiotherapy after esophagectomy have double survival time, time to recurrence, and recurrence-free survival, compared with patients treated with esophagectomy alone. The purpose of using radiotherapy and/or chemotherapy is to reduce the rate of locoregional recurrence and/or distant metastasis and enhance the disease-free survival time and overall survival time. Thus, we aimed to develop a statistical tool to predict the probability of locoregional recurrence or distant metastasis in esophageal cancer patients after radical esophagectomy.

Nomograms are widely used to help doctors make decisions [6]. Nomograms are frequently used not only for predicting survival in patients with all types of cancer but also for successfully quantifying risk prediction according to clinicopathological variables [7]. Instead of associating a risk factor with a hazard ratio, the nomogram integrates the factor along with the other predictive factors to assess the individual patient’s absolute risk [8]. To the best of our knowledge, however, there is no study to predict the risk of locoregional recurrence or distant metastasis for patients with esophageal cancer after surgery.

The primary aim of this study was to create a prediction model for the risk of locoregional recurrence or distant metastasis in esophageal cancer patients who were treated with esophagectomy and regional lymphadenectomy.

## Methods

At total of 408 esophageal cancer patients who underwent esophagectomy and regional lymphadenectomy at the First Affiliated Hospital of Wenzhou Medical University during the period from January 2006 through December 2012 were retrospectively reviewed. The eligibility criteria for review were as follows: 1) underwent esophagectomy and regional lymphadenectomy; 2) esophageal cancer proven by pathology; 3) no residual tumor (R0); and 4) no distant metastasis (M1) before surgery. The surgical approaches of radical esophagectomy consisted of a right thoracotomy with 3-field lymphadenectomy and gastric tube reconstruction. 32 patients received the neoadjuvant chemotherapy regimen consisting of intravenous Paclitaxel (135 mg/m^2^ on day 1 of each cycle) plus intravenous Cisplatin (25 mg/m^2^ on days 1–3 of each cycle). 22 patients received the neoadjuvant chemotherapy regimen consisting of intravenous Fluorouracil (750 mg/m^2^ on day 1–3 of each cycle) plus intravenous Cisplatin (25 mg/m^2^ on days 1–3 of each cycle). 65 patients received the adjuvant chemotherapy regimen consisting of intravenous Docetaxel (75 mg/m^2^ on day 1 of each cycle) plus intravenous Cisplatin (25 mg/m^2^ on days 1–3 of each cycle). 42 patients received the adjuvant chemotherapy regimen consisting of intravenous Paclitaxel (135 mg/m^2^ on day 1 of each cycle) plus intravenous Cisplatin (25 mg/m^2^ on days 1–3 of each cycle). 11 patients received the adjuvant chemotherapy regimen consisting of intravenous Fluorouracil (750 mg/m^2^ on day 1–3 of each cycle) plus intravenous Cisplatin (25 mg/m^2^ on days 1–3 of each cycle). 14 patients received several otherchemotherapy regimen.The study was approved by the Medical Ethical Committee of the First Affiliated Hospital of Wenzhou Medical University. All patients were given informed written consent.

### Follow-up

Every patient was seen postoperatively at three-month intervals for the first two years, at six-month intervals for three years and annually thereafter. The follow-up visits consisted of a physical examination, blood count examination, hepatic and renal function, neck CT scanning, chest CT scanning, whole abdomen CT scanning, and endoscopic examination.

### Statistical analyses

Univariate and multivariate COX regression analyses were used to test the association between the clinicopathologic characteristics (including sex, age, tumor length, tumor width, tumor site, pathological type, differentiation, T-staging, N-staging, lymph vascular space invasion (LVSI), perineural invasion (PNI) and adjuvant chemotherapy) and the risk of locoregional recurrence or distant metastasis. Locoregional recurrence included recurrence of anastomotic stoma and the tumor bed which included primary lesion before operation and regional lymph node area. Distant metastasis included lymphatic metastasis and organ metastasis. Tumor length and width were measured from the resected tissue by the naked eye. The receiver-operating characteristic (ROC) curve analysis was performed to calculate the area under the curve (AUC) and check the value of the statistically significant variables (*P* < 0.05). The risk of locoregional recurrence or distant metastasis nomograms was based on multivariate COX regression models. Statistically significant variables (*P* < 0.05) from the multivariate COX regression analysis were entered into the nomograms. Calibration curves were plotted to assess the agreement between the actual rate of locoregional recurrence or distant metastasis and the predicted probabilities of locoregional recurrence or distant metastasis. The discrimination of the nomograms for predicting locoregional recurrence or distant metastasis was assessed by the concordance index (C-index). Univariate and multivariate COX regression analyses were performed with SPSS 18.0. The nomograms and calibration curves were conducted using R version 2.8.1 (R foundation for Statistical Computing) with the rms package.

## Results

The patient characteristics are shown in Table 1. In total, 357 (87.5%) patients were male, and the median age of all the patients was 59.5 years old. A total of 376 (92.2%) of the tumors were squamous carcinoma, 169 (41.4%) patients were treated with adjuvant chemotherapy, 186 patients developed locoregional recurrence, 210 patients developed distant metastasis, 82 patients developed both locoregional recurrence and distant metastasis, and 94 patients did not develop locoregional recurrence or distant metastasis.

**Table 1:** Patient characteristics

Characteristic	No. of patients(%)
Total	408
Sex	
Female	51(12.5%)
Male	357(87.5%)
Age	
Median	59.5 years
Range	39–83
Tumor length(cm)	
Median	4 cm
Range	0.3–10.5 cm
Tumor width(cm)	
Median	2 cm
Range	0.3-9 cm
Tumor site	
Upper thoracic portion	49(12.0%)
Middle thoracic portion	208(51.0%)
Lower thoracic portion	151(37.0%)
Pathological type	
Squamous carcinoma	376(92.2%)
Adenocarcinoma	29(7.1%)
Other	3(0.7%)
Differentiation	
Low	112(27.5%)
Middle	204(50.0%)
High	92(22.5%)
T-staging	
T1	67(16.4%)
T2	106(26.0%)
T3	235(57.6%)
N-staging	
N0	205(50.2%)
N1	116(28.4%)
N2	45(11.0%)
N3	42(10.4%)
LVSI	
Positive	85(20.8%)
Negative	323(79.2%)
PNI	
Positive	72(17.6%)
Negative	336(82.4%)
Adjuvant chemotherapy	
Yes	168(41.2%)
No	240(58.8%)

*LVSI* lymph vascular space invasion, *PNI* perineural invasion

**Fig. 1:**
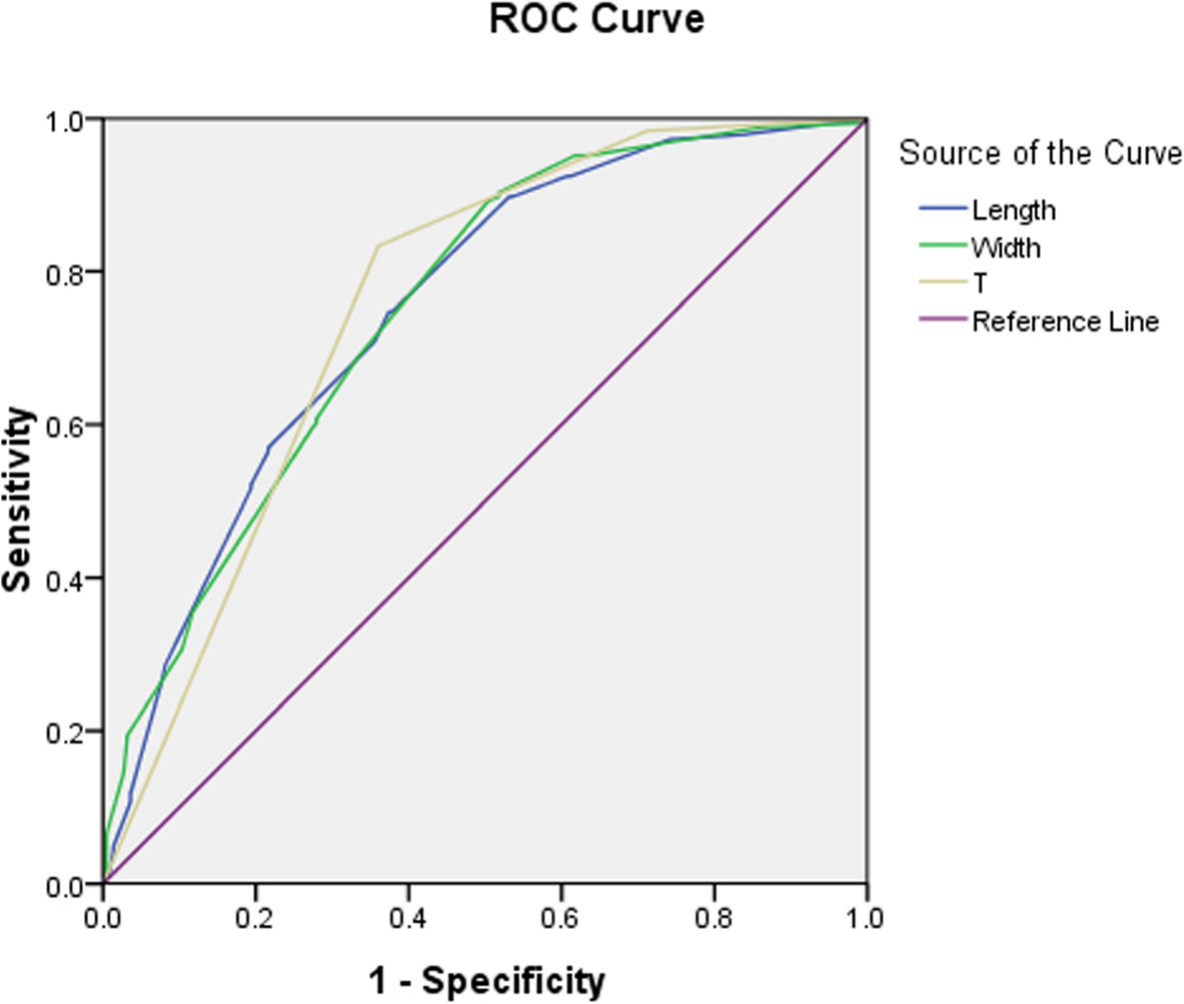
Receiver operating characteristic (ROC) curve plotted to check the value of statistically significant variables in the COX regression model for predicting risk of locoregional recurrence

**Fig. 2:**
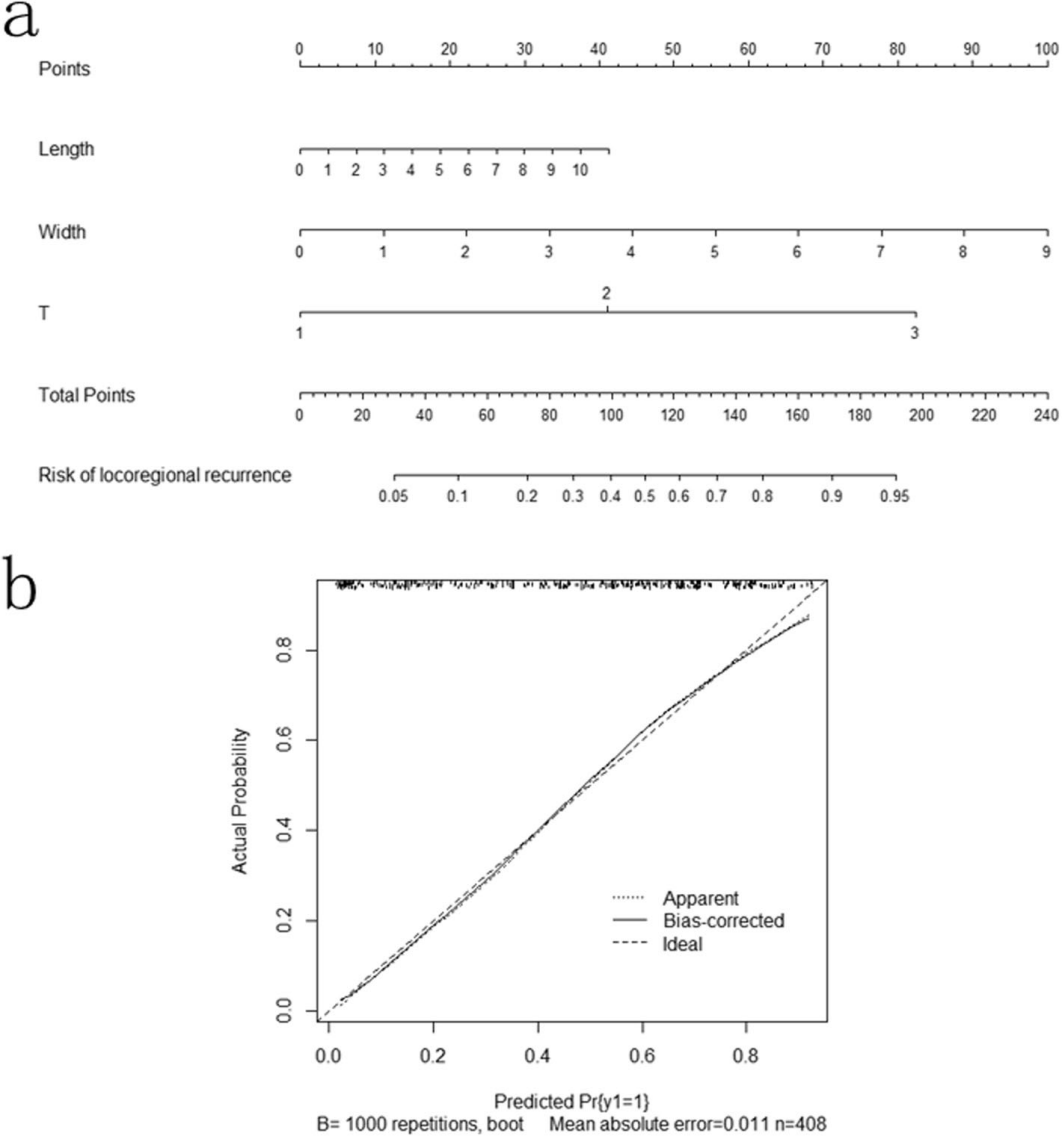
**a** Nomograms for predicting risk of locoregional recurrence in esophageal cancer patients after radical esophagectomy. The units of length and width are centimeters (cm). **b** Calibration curve for risk of locoregional recurrence nomogram.

The median follow-up was 49.4 months (range: 1.5 to 148.2 months). The univariate COX regression analyses revealed that tumor length, tumor width, T-staging and PNI were significantly associated with locoregional recurrence. The multivariate COX regression analyses revealed that tumor length (hazard ratio(HR) 1.225 [95% CI 1.079–1.391], *P* = 0.002), tumor width (HR 1.150 [95% CI 1.001–1.320], *P* = 0.048) and T-staging (HR 3.048 [95% CI 2.182–4.256], *P* < 0.001) were predictors of the risk of locoregional recurrence. Table 2 show the univariate COX regression model for predicting the risk of locoregional recurrence. According to the ROC analysis, the AUC of the tumor length, tumor width and T-staging were 0.751, 0.752, and 0.755, respectively. The ROC curve is shown in Fig. 1. Figure 2a shows the nomogram built from this model for predicting the risk of locoregional recurrence. The bias-corrected curve showed no significant departure from the ideal curve in Fig. 2b. The mean absolute error was 1.1%. The C-index of this nomogram for predicting the locoregional recurrence was 0.83 (95% confidence interval (CI), 0.75–0.91).

**Table 2:** Univariate analyses and multivariate analyses of locoregional recurrence

	Univariate			Multivariate		
Variable	HR	95%CI	*P*	HR	95%CI	*P*
Sex	1.023	0.666–1.571	0.916	–	–	–
Age	1.007	0.990–1.024	0.445	–	–	–
Tumor length	1.460	1.359–1.568	< 0.001	1.225	1.079–1.391	0.002
Tumor width	1.514	1.401–1.636	< 0.001	1.150	1.001–1.320	0.048
Tumor site	1.056	0.841–1.327	0.640	–	–	–
Pathological type	1.429	0.932–2.191	0.102	–	–	–
Differentiation	0.862	0.705–1.055	0.150	–	–	–
T-staging	4.006	2.898–5.539	< 0.001	3.048	2.182–4.256	< 0.001
N-staging	1.139	0.993–1.307	0.063	–	–	–
LVSI	1.321	0.939–1.858	0.110	–	–	–
PNI	1.561	1.096–2.223	0.014	1.289	0.901–1.842	0.165
Adjuvant chemotherapy	1.091	0.815–1.459	0.559	–	–	–

*LVSI* lymph vascular space invasion, *PNI* perineural invasion

**Fig. 3:**
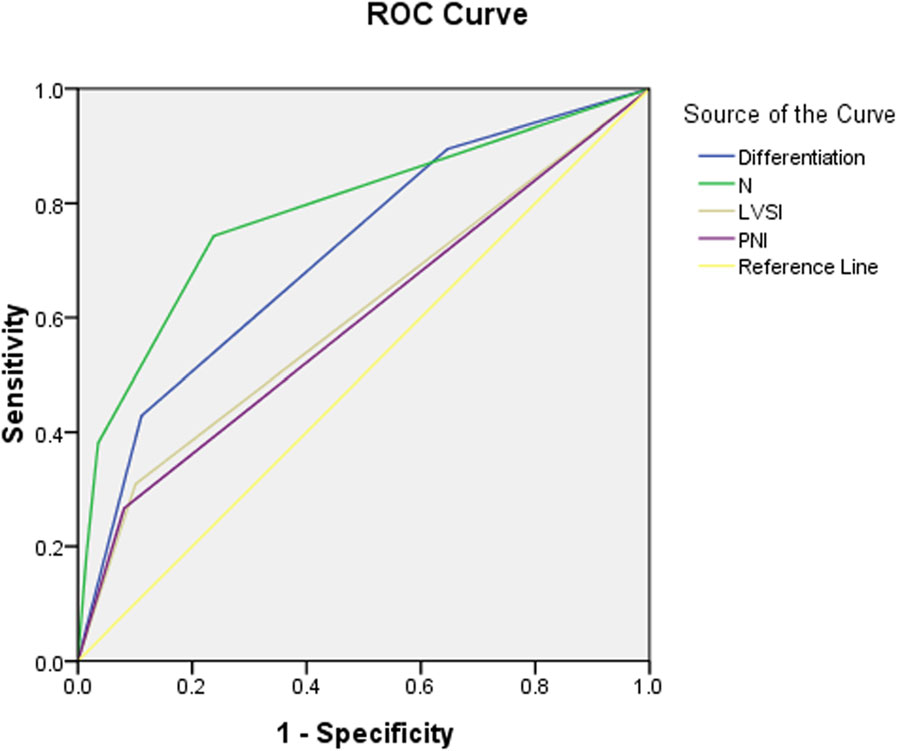
Receiver operating characteristic (ROC) curve plotted to check the value of statistically significant variables in the COX regression model for predicting risk of distant metastases

**Fig. 4:**
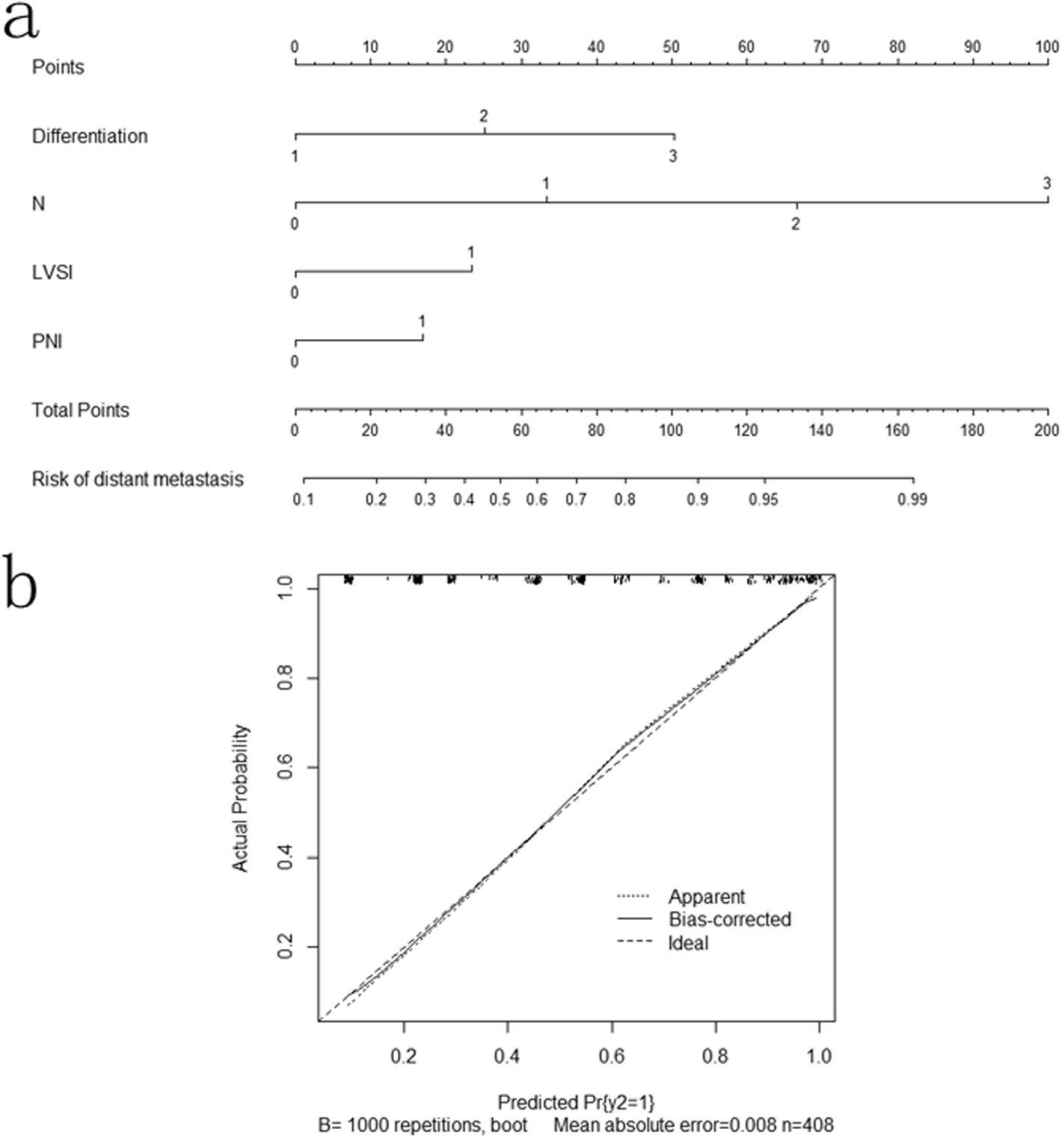
**a** Nomograms for predicting risk of distant metastases in esophageal cancer patients after radical esophagectomy. Differentiation: 1 = high differentiation; 2 = middle differentiation; 3 = low differentiation. LVSI, lymph vascular space invasion: 0 = negative(−); 1 = positive(+). PNI, perineural invasion: 0 = negative(−); 1 = positive(+). **b** Calibration curve for risk of distant metastases nomogram.

In the univariate COX regression analyses, tumor length, tumor width, differentiation, T-staging, N-staging, LVSI, PNI and adjuvant chemotherapy were significantly associated with distant metastasis. In the multivariate COX regression analyses, the risk of distant metastasis was positively associated with differentiation (HR 1.831 [95% CI 1.461–2.296], *P* < 0.001), N-staging (HR 1.558 [95% CI 1.366–1.778], P < 0.001), LVSI (HR 1.416 [95% CI 1.037–1.935], *P* = 0.029) and PNI (HR 1.598 [95% CI 1.153–2.214], *P* = 0.005). Table 3 show the univariate COX regression model for predicting the risk of distant metastasis. According to the ROC analysis, the AUC of differentiation, N-staging, LVSI and PNI was 0.713, 0.785, 0.604, and 0.593, respectively. The ROC curve is shown in Fig. 3. Figure 4a shows the nomogram built from this model for predicting the risk of distant metastasis. The bias-corrected curve showed no significant departure from the ideal curve in Fig. 4b. The mean absolute error was 0.8%. The C-index of this nomogram for predicting distant metastasis was 0.87 (95% CI, 0.79–0.94).

**Table 3:** Univariate analyses and multivariate analyses of distant metastasis

	Univariate			Multivariate		
Variable	HR	95%CI	*P*	HR	95%CI	*P*
Sex	0.713	0.454–1.119	0.142	–	–	–
Age	0.985	0.970–1.001	0.064	–	–	–
Tumor length	1.129	1.054–1.210	0.001	1.089	0.966–1.228	0.162
Tumor width	1.136	1.034–1.248	0.008	0.949	0.820–1.097	0.477
Tumor site	1.164	0.938–1.446	0.168	–	–	–
Pathological type	1.058	0.684–1.636	0.801	–	–	–
Differentiation	2.354	1.911–2.901	< 0.001	1.831	1.461–2.296	< 0.001
T-staging	1.606	1.311–1.968	< 0.001	1.136	0.897–1.438	0.289
N-staging	1.934	1.720–2.176	< 0.001	1.558	1.366–1.778	< 0.001
LVSI	2.515	1.874–3.375	< 0.001	1.416	1.037–1.935	0.029
PNI	2.774	2.039–3.774	< 0.001	1.598	1.153–2.214	0.005
Adjuvant chemotherapy	2.274	1.731–2.998	< 0.001	1.341	0.997–1.803	0.052

*LVSI* lymph vascular space invasion, *PNI* perineural invasion

## Discussion

After radical surgery, cancer progression is characterized as either a locoregional recurrence or a distant metastasis, and sometimes it includes both. The accuracy of predicting the locoregional recurrence risk and distant metastasis risk is increasingly important for making adjuvant radiotherapy and/or adjuvant chemotherapy recommendations for esophageal cancer patients after radical esophagectomy. Thus, we developed a statistical tool for predicting the risk of locoregional recurrence or distant metastasis. A nomogram is a statistical tool that provides a risk prediction for an individual patient. The aim of this nomogram was to discriminate patients with a high risk of locoregional recurrence from those with a low risk of locoregional recurrence, or patients with a high risk of distant metastasis from those with a low risk of distant metastasis. This method should provide the oncologist with advice on whether an esophageal cancer patient, after radical operation, should be treated with adjuvant treatment (chemotherapy, radiotherapy or chemoradiotherapy) or not.

In this study, we found that in the multivariate COX regression analyses, tumor length, tumor width and T-staging were significant risk predictors of locoregional recurrence in esophageal cancer patients undergoing radical esophagectomy, while differentiation, N-staging, LVSI and PNI were significant risk predictors of distant metastasis in those patients. Therefore, tumor length, tumor width and T-staging were selected to build a nomogram to predict the risk of locoregional recurrence, and differentiation, N-staging, LVSI and PNI were selected to build other nomogram to predict the risk of distant metastasis.

Lerut T et al. [9] reported that cox-regression analyses identified the presence of complications, extracapsular lymph node involvement, and R1-status as significant determinators of recurrence for esophageal cancer after transthoracic esophagectomy. Chen GP et al. [10] reported that age, sex, T-staging, N-staging and red cell distribution width were prognostic factors in a nomogram predicting the survival risk for patients with esophageal squamous cell carcinoma after radical esophagectomy. Liu JS and colleagues [11] developed a nomogram predicting survival based on T-staging, N-staging, Glasgow Prognostic Score, platelet lymphocyte ratio and lymphocyte monocyte ratio in patients with esophageal squamous cell carcinoma who underwent radical esophagectomy. Gertler R et al. [12] reported that the grade of tumor differentiation, lymphovascular invasion, patient age, depth of tumor invasion and multifocal tumor revealed were independent predictive factors for the probability of lymph node metastases in patients with pT1 carcinomas after primary surgery for esophageal cancer, esophagogastric junction cancer, and stomach cancer. Meanwhile, a nomogram established was based on these factors [12]. Lin SH and colleagues [13] used gender, tumor grade, standardized uptake value of the primary tumor by positron emission tomography (PET) after chemoradiation, baseline T status (by endoscopic sonography) and esophagogastroduodenoscopy biopsy results after chemoradiation as predictive factors in a nomogram for predicting pathologic complete response in esophageal cancer patients receiving chemoradiation without surgery. Duan J et al. [14] reported that two nomograms, based on tumor length, gender, N stage, T stage and chemotherapy cycles, were created for predicting 1-, 2-, 3-year disease-free survival and 1-, 2-, 3-year overall survival in esophageal squamous cell carcinoma patients who underwent radical esophagectomy and adjuvant chemotherapy. Suzuki A et al. [15] reported that two nomograms were established to predict 3-, 5-year disease-free survival and 3-, 5-year overall survival in patients with esophageal and gastroesophageal junction cancers receiving definitive chemoradiotherapy according to five factors, including stage, grade, histology, initial standardized uptake value of PET and clinical complete response. Our study was the first attempt to use COX regression analyses and to develop and validate a predictive nomogram to predict the risk of locoregional recurrence and the risk of distant metastasis for patients with esophageal cancer after radical esophagectomy.

The adjuvant treatment decision was made based on this method after surgery, thus avoiding the use of chemotherapy in those patients who might have low risk in distant metastasis and the use of radiotherapy in those patients who might have low risk in locoregional recurrence. Adelstein DJ et al. [16] reported that the 4-year overall survival in resected esophageal cancer patients with a pathologic stage of T3, N1, or M1a receiving concurrent chemoradiotherapy is 51%, distant metastatic control is 56%, and locoregional control is 86%. The Japanese Esophageal Oncology Group [17] reported that there was no significant difference in survival up to 5 years in 2 groups that were treated with chemotherapy or radiotherapy after surgery. The reason that there was no significant difference in survival up to 5 years in the 2 groups was that there was no difference in the distribution of the risk of locoregional recurrence and distant metastasis in the 2 groups. Ando N et al. [18] demonstrated that a patient with pN0 or pN1 did not need adjuvant chemotherapy, which leads to complications, such as vomit, diarrhea, granulocytopenia, and reduced quality of life. The aim of this study was to avoid the use of unnecessary radiotherapy or chemotherapy when treating resected esophageal cancer patients with a low risk of locoregional recurrence or distant metastasis.

However, there were some limitations in this study. Firstly, the data from only single institution were involved. It may limit the applicability of this two nomograms. Secondly, the sample size of our study was limited. Thirdly, this was a risk assessment model and thus suffers the limitation associated with model nature. For example, it is difficult to accurately capture the complexities of change in patients’ condition. Consequently, further multi-institutional investigation is needed to generate more accurate and representative risk model.

## Conclusion

The two nomograms in this study were developed to predict the risk of locoregional recurrence and distant metastasis in esophageal cancer patients after radical esophagectomy. These nomograms could be used to provide advice for patients on whether they need to receive adjuvant therapy or not.

## Abbreviations

AUC: Area under the curve
CI: Confidence interval
C-index: Concordance index
HR: Hazard ratio
LVSI: Lymph vascular space invasion
PET: Positron emission tomography
PNI: Perineural invasion
ROC: Receiver-operating characteristic

## References

[1] Khushalani N (2008). Cancer of the esophagus and stomach. Mayo Clin Proc.

[2] Pasquer A, Gronnier C, Renaud F, Duhamel A, Théreaux J, Carrere N (2015). Impact of adjuvant chemotherapy on patients with lymph node-positive esophageal Cancer who are primarily treated with surgery. Ann Surg Oncol.

[3] Brescia AA, Broderick SR, Crabtree TD, Puri V, Musick JF, Bell JM (2016). Adjuvant therapy for positive nodes after induction therapy and resection of esophageal Cancer. Ann Thorac Surg.

[4] Bédard EL, Inculet RI, Malthaner RA, Brecevic E, Vincent M, Dar R (2001). The role of surgery and postoperative chemoradiation therapy in patients with lymph node positive esophageal carcinoma. Cancer.

[5] Rice TW, Adelstein DJ, Chidel MA, Rybicki LA, DeCamp MM, Murthy SC (2003). Benefit of postoperative adjuvant chemoradiotherapy in locoregionally advanced esophageal carcinoma. J Thorac Cardiovasc Surg.

[6] Rouzier R, Pusztai L, Garbay JR, Delaloge S, Hunt KK, Hortobagyi GN (2006). Development and validation of nomograms for predicting residual tumor size and the probability of successful conservative surgery with neoadjuvant chemotherapy for breast cancer. Cancer.

[7] Wang Y, Li J, Xia Y, Gong R, Wang K, Yan Z (2013). Prognostic nomogram for intrahepatic cholangiocarcinoma after partial hepatectomy. J Clin Oncol.

[8] Rudloff U, Jacks LM, Goldberg JI, Wynveen CA, Brogi E, Patil S (2010). Nomogram for predicting the risk of local recurrence after breast-conserving surgery for ductal carcinoma in situ. J Clin Oncol.

[9] Lerut T, Moons J, Coosemans W, Van Raemdonck D, De Leyn P, Decaluwé H (2009). Postoperative complications after transthoracic esophagectomy for cancer of the esophagus and gastroesophageal junction are correlated with early cancer recurrence: role of systematic grading of complications using the modified Clavien classification. Ann Surg.

[10] Chen GP, Huang Y, Yang X, Feng JF (2015). A Nomogram to predict prognostic value of red cell distribution width in patients with esophageal Cancer. Mediat Inflamm.

[11] Liu JS, Huang Y, Yang X, Feng JF (2015). A nomogram to predict prognostic values of various inflammatory biomarkers in patients with esophageal squamous cell carcinoma. Am J Cancer Res.

[12] Gertler R, Stein HJ, Schuster T, Rondak IC, Höfler H, Feith M (2014). Prevalence and topography of lymph node metastases in early esophageal and gastric cancer. Ann Surg.

[13] Lin SH, Wang J, Allen PK, Correa AM, Maru DM, Swisher SG (2015). A nomogram that predicts pathologic complete response to neoadjuvant chemoradiation also predicts survival outcomes after definitive chemoradiation for esophageal cancer. J Gastrointest Oncol.

[14] Duan J, Deng T, Ying G, Huang D, Zhang H, Zhou L (2016). Prognostic nomogram for previously untreated patients with esophageal squamous cell carcinoma after esophagectomy followed by adjuvant chemotherapy. Jpn J Clin Oncol.

[15] Suzuki A, Xiao L, Hayashi Y, Blum MA, Welsh JW, Lin SH (2012). Nomograms for prognostication of outcome in patients with esophageal and gastroesophageal carcinoma undergoing definitive chemoradiotherapy. Oncology.

[16] Adelstein DJ, Rice TW, Rybicki LA, Saxton JP, Videtic GM, Murthy SC (2009). Mature results from a phase II trial of postoperative concurrent chemoradiotherapy for poor prognosis cancer of the esophagus and gastroesophageal junction. J Thorac Oncol.

[17] Japanese Esophageal Oncology Group (1993). A comparison of chemotherapy and radiotherapy as adjuvant treatment to surgery for esophageal carcinoma. Chest.

[18] Ando N, Iizuka T, Ide H, Ishida K, Shinoda M, Nishimaki T (2003). Surgery plus chemotherapy compared with surgery alone for localized squamous cell carcinoma of the thoracic esophagus: a Japan clinical oncology group study--JCOG9204. J Clin Oncol.

